# New Insights on the Use of Dietary Polyphenols or Probiotics for the Management of Arterial Hypertension

**DOI:** 10.3389/fphys.2016.00448

**Published:** 2016-10-06

**Authors:** José L. de Brito Alves, Vanessa P. de Sousa, Marinaldo P. Cavalcanti Neto, Marciane Magnani, Valdir de Andrade Braga, João H. da Costa-Silva, Carol G. Leandro, Hubert Vidal, Luciano Pirola

**Affiliations:** ^1^Department of Nutrition, Health Sciences Center, Federal University of ParaíbaJoão Pessoa, Brazil; ^2^CarMeN (Cardio, Metabolism, Diabetes and Nutrition) Laboratory, Institut National de la Santé et de la Recherche Médicale U1060, INRA 1397, Université Claude Bernard Lyon 1Oullins, France; ^3^Departments of Clinical Analyses, Toxicology and Food Sciences, University of São PauloRibeirão Preto, Brazil; ^4^Department of Food Engineering, Technology Center, Federal University of ParaíbaJoão Pessoa, Brazil; ^5^Biotechnology Center, Federal University of ParaíbaJoão Pessoa, Brazil; ^6^Department of Physical Education and Sport Sciences, Federal University of PernambucoVitoria de Santo Antão, Brazil

**Keywords:** probiotics, hypertension, blood pressure, dietary polyphenols

## Abstract

Arterial hypertension (AH) is one of the most prevalent risk factors for cardiovascular diseases (CD) and is the main cause of deaths worldwide. Current research establish that dietary polyphenols may help to lower blood pressure (BP), thus contributing to the reduction of cardiovascular complications. In addition, the health benefits of probiotics on BP have also attracted increased attention, as probiotics administration modulates the microbiota, which, by interacting with ingested polyphenols, controls their bioavalability. The aim of the present mini-review is to summarize and clarify the effects of dietary polyphenols and probiotics administration on BP using combined evidence from clinical and experimental studies, as well as to discuss the current debate in the literature about the usefulness of this nutritional approach to manage BP. Clinical trials and experimental studies have demonstrated that consuming dietary polyphenols or probiotics in adequate amounts may improve BP, ranging from modest to greater effects. However, the mechanisms linking probiotic intake and reduced BP levels need to be further elucidated as a definitive consensus on the link between intake of polyphenols or probiotics and improvement of AH has not been reached yet.

## Introduction

Arterial hypertension (AH) affects more than 1 billion people and is the major risk factor for CD (Hedner et al., 2012). The cause of AH has been difficult to identify due to its multi-factorial nature, which involves genetic and environmental factors. A balanced and healthy diet plays a key role in the maintenance of the cardiovascular health status, which is a major determinant of lifespan. In the last decades, in both western and developing countries, the consumption of highly caloric—rich in fat and carbohydrates—and sodium-rich diets have become predominant, increasing the incidence of AH (Bjerregaard, 2010; Popkin, 2011).

The regulation of BP is one of the most complex physiological functions and depends on the integrated actions of cardiovascular, renal, neural and endocrine systems (Corry and Tuck, 1999; de Brito Alves et al., 2015). In addition, augmentation of proinflammatory markers, reactive oxygen species and dysfunction in energy metabolism are related to hypertensive conditions (Carthy, 2014).

Despite the advances in the understanding of the pathophysiology and pharmacotherapy of AH, interventional strategies helping to reduce BP levels remain one of the great problems to be developed. Studies have investigated the beneficial effect of dietary changes on BP levels and also identified a BP-lowering activity on different dietary compounds.

The intestine and its microbiota constitute an important site of interaction with the dietary compounds (Bäckhed et al., 2004), and in particular polyphenols, whose bioavailability is dependent on prior metabolization by the microbiota (Ozdal et al., 2016; Stevens and Maier, 2016). The interaction between gut microbiota and diet can affect the gut-immune homeostasis, cell proliferation, metabolism, and intestinal permeability (Ding et al., 2010). Growing evidence supports the notion that the gut microbiota plays an important role in the development of CD and AH (Khalesi et al., 2014; Jose and Raj, 2015), and dietary compounds might modulate the gut microbiota favoring or lowering the BP (Buettner et al., 2007; Anhê et al., 2015).

Accordingly, findings have suggested the notion that dietary polyphenols and probiotic supplementation could help alleviating AH, by altering the gut microbiota and favoring an antioxidant activity (Anhê et al., 2015; Gómez-Guzmán et al., 2015; Grosso et al., 2016), although null and contrary findings have also been reported (Ras et al., 2013). In the present mini-review, we summarize the current understanding emerging from experimental, clinical and epidemiological studies, on how dietary polyphenols and probiotics intake may help to lower BP in hypertensive conditions.

## Dietary polyphenols and the control of BP

Polyphenols are a large and heterogeneous family of bioactive molecules found in numerous food sources. Generally defined as dietary antioxidants, polyphenols have been established as bioactive compounds that benefit human health via modulation of metabolism (Choi et al., 2014). Dietary polyphenols are mainly classified in catechins (proanthocyanidins), flavonols, flavanones, ellagitannins, and isoflavones. Studies have investigated the effects of dietary polyphenols, or their metabolites supplementation either via administration as polyphenol-enriched diets, polyphenol extracts from foods or as administration of specific polyphenolic compounds (e.g., quercetin, rutin, resveratrol, hesperidin, cinnamon) (Mendes-Junior et al., 2013; Amiot et al., 2016).

Dietary polyphenols have been shown to exert beneficial effects on markers for cardiovascular risk factors, including reduction of BP, improvement of endothelial function and lowering of plasma lipids. Mechanistically, it has been suggested that dietary polyphenols can alleviate hypertension through anti-inflammatory and anti-oxidant effects, and increased oxide nitric (NO) production (Davinelli and Scapagnini, 2016). The anti-inflammatory effect is associated with a reduced expression of the redox-sensitive nuclear factor-kB (NF-κB), while that the anti-oxidant effect of polyphenols is related to improved enzymatic activities of superoxide dismutase, catalase and glutathione peroxidase. In addition, polyphenols participate in the activation of the redox-sensitive phosphoinositide 3 (PI3)-kinase/Akt pathway, leading to increased formation of NO (Davinelli and Scapagnini, 2016). Taken together, all these pathways help to reduce blood pressure in hypertensive conditions.

Other studies have, however, reached negative or null effects. These effects have been investigated in several cohorts and randomized clinical trials (Table 1).

**Table 1:** **Summary of the major studies investigating the relationship between dietary polyphenols intake and blood pressure/cardiovascular health**.

**Study acronym (or 1^st^ author)**	**Study name**	**Number of participants**	**Intervention**	**Primary endpoint observed**	**Other parameters**	**References**
PREDIMED	Prevention with Mediterranean diet	7447	2 different polyphenol-rich diets	Decreased rate of major cardiovascular events	Decreased systolic and diastolic blood pressure	Estruch et al., 2013; Medina-Remón et al., 2016
HAPIEE	Health, Alcohol and Psychosocial factors In Eastern Europe cohort study	8821(Polish cohort)	Observational study	Amelioration of metabolic syndrome parameters (Body mass index, waist circumference, blood pressure, triglycerides) in polyphenol-rich diets		Grosso et al., 2016
EPIC	European Prospective Investigation into Cancer and Nutrition	20,343 (Greek cohort)	Observational study with evaluation of a “Mediterranean Diet Score”	Inverse correlation between olive oil intake and arterial blood pressure		Psaltopoulou et al., 2004
Ras et al.	Effect of polyphenol-rich grape seed extract on ambulatory blood pressure in subjects with pre- and stage I hypertension	35	Administration of polyphenol-rich grape seed extract in a double-blind, placebo controlled, randomized, parallel-group trial	No lowering of blood pressure in untreated subjects with pre- and stage I hypertension		Ras et al., 2013
Mogollon et al.	Blood pressure and endothelial function in healthy, pregnant women after acute and daily consumption of flavanol-rich chocolate: a pilot, randomized controlled trial	23	Randomized Controlled trialof acute and chronic consumption of flavanol-rich chocolate	No association with significant changes in systolic or diastolic blood pressure		Mogollon et al., 2013

In a small-scale randomized nutritional trial, the administration of a polyphenol-rich diet (approximately 3000 mg polyphenol/day) reduced postprandial triglyceride-rich lipoprotein plasma concentrations and oxidative stress in study participants with a high risk of CD (Annuzzi et al., 2014).

The beneficial effects of polyphenol supplementation have also been demonstrated in the larger PREDIMED (Prevention with Mediterranean Diet) cohort. In this study, the consumption of a Mediterranean diet—supplemented with extra-virgin olive oil or nuts—resulted in reduced incidence of cardiovascular events (myocardial infarction, stroke, or death from cardiovascular causes) (Estruch et al., 2013). Within the PREDIMED cohort, a sub-study on 1139 high-risk participants was performed in which two different polyphenol-rich diets (based on supplementation with extra-virgin olive oil or nuts) were randomly assigned. The increase in polyphenol intake—which was unequivocally identified as increased total urinary polyphenol excretion—was associated with decreased inflammatory biomarkers and a decrease of systolic and diastolic BP (Medina-Remón et al., 2016).

In the Polish population of the non-interventional HAPIEE cohort (Health, Alcohol and Psychosocial factors In Eastern Europe), elevated dietary intake of polyphenols was associated with lower body mass index (BMI), waist circumference (WC), BP and triglycerides, further suggesting that high polyphenols intake is inversely associated to metabolic syndrome and its clinical manifestations (Grosso et al., 2016). To ascertain whether olive oil polyphenols alleviate AH independently from the lipid component of olive oil, which is rich in monounsaturated fatty acids (MUFA), a double-blind, crossover dietary-intervention study was performed in which mildly hypertensive women received polyphenol-rich olive oil (approximately 30 mg/day) in a first dietary period, and polyphenol-free olive oil in a second dietary period. Interestingly, only polyphenol-rich olive oil decreased BP and improved endothelial function, underscoring the specific role of polyphenols within the olive oil (Moreno-Luna et al., 2012). In addition, in a randomized, double blind, controlled, crossover trial, the hypotensive and lipid-lowering capacity have also been demonstrated for olive leaf extract (Lockyer et al., 2015, 2016).

Although the studies presented above indicate that administration of dietary polyphenols might contribute to the control of BP, a definitive consensus has not been reached yet, as independent investigations did not support the hypothesis that dietary intake of polyphenols is beneficial to cardiovascular health (Ras et al., 2013).

## Resveratrol

Resveratrol (3,5,4′-trihydroxy-trans-stilbene) is a stilbenoid. Studies demonstrated that resveratrol possess an intrinsic cancer chemopreventive activity (Jang et al., 1997), and further interest on this molecule arose with the identification of its capability to increase lifespan when administered to experimental organisms including *Saccharomyces cerevisiae, Caenorhabditis elegans, Drosophila melanogaster* (Ingram et al., 2006), and mice (Baur et al., 2006). Furthermore, it was soon documented in animal models that resveratrol (i) protects from the development of obesity-dependent metabolic disorders (Fröjdö et al., 2008) and (ii) can be the molecule responsible for the cardio-protective effects of red wine (Wu et al., 2011).

Preclinical studies have investigated the effects of resveratrol administration on cardiovascular health. In spontaneously hypertensive rats (SHR) and in mice rendered hypertensive by angiotensin-II injection, resveratrol administration reduced oxidative stress in the endothelium, improved vascular function and attenuated AH (Dolinsky et al., 2013). Resveratrol supplementation to SHR dams during the perinatal period (from gestation to weaning of the offspring) alleviated the development of AH in the offspring at the adult age (Care et al., 2016). These finding have prompted the development of several small-scale clinical trials evaluating the effects of resveratrol supplementation on cardiovascular systems.

In some trials, resveratrol supplementation (range 200–300 mg/day) improved insulin resistance, glycemic profile and lipid metabolism (Wong et al., 2011; Chen et al., 2015). However, in other studies, 12-week supplementation with 500 mg/day resveratrol (Faghihzadeh et al., 2015) or 150 mg/day for 4 weeks (van der Made et al., 2015) did not change metabolic risk markers related to cardiovascular dysfunction.

A recent meta-analysis of 10 randomized clinical trials failed to show any benefit of resveratrol supplementation on cardiovascular risk factors. In particular resveratrol had no effect on systolic or diastolic BP (Sahebkar et al., 2015). However, an independent meta-analysis performed on more stringent adjudication criteria for study quality, including only six studies comprising a total of 247 subjects, suggested that resveratrol consumption decreased systolic BP (at the higher administered doses) while having no significant effects on diastolic BP (Liu et al., 2015). Given the absence of a clear consensus on the effects on resveratrol on the control of BP in humans, as opposed to the clear conclusions in rodent models, the need for larger and well-designed clinical trials, was solicited by the authors of both meta-analysis to definitely prove, or reject, a causal link between resveratrol administration and the control of BP (Novelle et al., 2015).

## Quercetin

Quercetin [2-(3,4-dihydroxyphenyl)-3,5,7-trihydroxy-4H-chromen-4-one] is a polyphenolic compound belonging to the class of flavonoids, which is naturally found in apples, berries, and red wine (Sigel et al., 1977). Epidemiological data and dietary analysis indicated that quercetin could contribute to decrease the risk of coronary heart disease in elderly patients (Hertog et al., 1993). To test the hypothesis that quercetin reduces BP in hypertensive patients, a randomized, double-blind, placebo-controlled, crossover study was performed to test the efficacy of quercetin supplementation (730 mg quercetin/day for 28 days) in pre-hypertensive and stage-1 hypertensive volunteers. Quercetin supplementation reduced systolic, diastolic and mean arterial pressure in stage-1 hypertensive volunteers, while having no effect on pre-hypertensive participants (Edwards et al., 2007). More recently, data obtained from hypertensive patients, also indicated that quercetin supplementation (162 mg/day for 6 weeks) exert cardio-protective effect, with a decrease of 24 h systolic BP of 3.6 mmHg (Brüll et al., 2015). On the other hand, a recent similar study in overweight-to-obese adults with AH did not detect changes in post-prandial BP, nor endothelial function, upon acute administration of quercetin (54 mg in a single dose) (Brüll et al., 2016). The effects of quercetin administration are likely dependent on the doses and administration time-span. A recent meta-analysis of randomized controlled trials investigating the effects of quercetin on BP support the idea that quercetin doses greater than 500 mg/day have a significant effect on the reduction of BP (Serban et al., 2016).

## Hesperidin

Hesperidin (30, 5, 9-dihydroxy-40-methoxy-7-orutinosyl flavanone) is an abundant flavonoid found in citrus fruit, particularly in the peel of oranges and lemon (Sharma et al., 2015). Reports suggest that hesperidin exerts a cardio-protective action via its antioxidant and antihypertensive properties (Wilmsen et al., 2005), as also demonstrated in an ischemic heart disease model in diabetic rats (Agrawal et al., 2014) and in SHR (Yamamoto et al., 2008; Ikemura et al., 2012). Over the last years, a large body of studies in cell culture and animal models has elucidated the molecular targets and mechanisms of action of hesperidin. Besides its cardio-protective actions, hesperidin has also shown anticancer, anti-inflammatory, and neuroprotective properties (Roohbakhsh et al., 2015). However, at present, clinical studies regarding the therapeutic effects of hesperidin have not appeared, and pre-clinical testing in humans is warranted to confirm the beneficial effects observed in animal models.

Recently it has been suggested that dietary polyphenols consumption could help maintain intestinal homeostasis and metabolic health. In light of recent discoveries, studies have shown that dietary polyphenols can exert part of their beneficial action through modulation of the microbiota (Anhê et al., 2016). For example, it was demonstrated that a polyphenol-rich cranberry extract prevented obesity and the metabolic syndrome in diet-induced obesity through prebiotic effect on the gut microbiota (Anhê et al., 2015). The investigation on whether dietary polypnehols attenuate BP through beneficial actions on gut microbiota is a newly developing field and the underlying mechanisms remain to be elucidated.

## Effects of probiotics on BP

The term probiotic means “for life” and it was first used to describe compounds produced by protozoa to stimulate the growth of other organisms (Lilly and Stillwell, 1965). Currently, the term probiotics refers to nonpathogenic microorganisms (bacteria or yeast) that, when ingested, are capable to reach the gut in sufficient amounts to confer health benefits (Parvez et al., 2006). Historically, probiotics derived from dairy products were the first to be isolated and studied (Tapsell, 2015). However, in the last decades, potentially probiotic strains from vegetable sources have also been isolated and their health benefits investigated (Rivera-Espinoza and Gallardo-Navarro, 2010; Vitali et al., 2012). Probiotics can be ingested either as supplements or incorporated in food or beverages in the form of dairy or non-dairy probiotic products (Jankovic et al., 2010; Vijaya Kumar et al., 2015). Despite the differences among the quantities of probiotic intake recommended by American or European Agencies to confer generic health claims, probiotic intake of around 10^6^–10^8^ CFU/g^−1^ (or mL^−1^) or 10^8^–10^10^ CFU/day (CFU, colony forming units) have shown to be efficacious (Champagne et al., 2011).

*Saccharomyces boulardii* is the main nonpathogenic yeast being used as probiotic. In addition, numerous bacteria belonging to the *Enterococcus, Pediococcus, Bacillus, Streptococcus, Lactococcus* and *Propionibacterium* genera are recognized as potential candidates for probiotic status. It is important to highlight that the *Lactobacillus (L.)* and *Bifidobacterium* (*B*.) genera constitute the majority of probiotics found on the market (Wohlgemuth et al., 2010; Champagne et al., 2011). Particularly, the lactic acid bacteria *L. acidophilus, L. casei, L. paracasei, L. fermentum, L. reuteri, L. plantarum, L. rhamnosus* and *L. salivarius*, as well as *B. bifidum, B. breve, B. infantis, B. lactis, B. longum* and *B. thermophilum* are among the main probiotics species marketed worldwide (Vijaya Kumar et al., 2015).

The benefits of the ingestion of probiotics by humans seem to be related to the improvement of the gut microbiota status, increase of enterocyte's resistance to pathogens, decrease or almost total elimination of pathogenic microorganisms within the intestinal tract, alleviation of nutritional intolerances (e.g., increased tolerance to lactose), enhancement of macro- and micro-nutrients bioavailability, and the reduction of the prevalence of allergies in susceptible individuals (Sharma and Devi, 2014).

Beneficial effects on intestinal permeability to macromolecules, including lipopolysaccharides and on gut inflammation are also considered as major mechanisms conferring health benefit to probiotics (Cani, 2014). A recent study in a germ-free mouse model has also shown that administration of probiotics belonging to the *Lactobacillus* genus may alleviate the negative effects of chronic under-nutrition on postnatal growth (Schwarzer et al., 2016).

Interestingly, experimental and clinical reports have demonstrated that improvement of the gut microbiota though probiotic supplementation might positively help in reducing BP in the hypertensive conditions (Ettinger et al., 2014; Jose and Raj, 2015; Mell et al., 2015).

Despite these findings, other reports has shown that the probiotic supplementation did not induce any significant alterations in BP, heart rate (HR) or cardiovascular risk markers, such as total cholesterol, low-density lipoprotein, pro-inflammatory markers (Barreto et al., 2014; Mahboobi et al., 2014; Ivey et al., 2015).

## Experimental findings

By using a hypertensive rat model treated with nitro-L-arginine methyl ester (L-NAME), it was demonstrated that the supplementation of fermented blueberries (very rich in polyphenols) containing *L. plantarum* (2 g/day for 4 weeks, containing 10^9^ CFU) reduced systolic (by approximately 45%) and diastolic (by approximately 45%) BP in hypertensive animals (Ahrén et al., 2015). Mechanistically, it has been suggested that fermentation of blueberries by *L. plantarum* could reduce BP trough a mechanism involving a nitric oxide (NO)-dependent pathway (Ahrén et al., 2015). However, another study showed that adding probiotics to a blueberry-enriched diet did not enhance, and actually might have impaired the anti-hypertensive effect of blueberry consumption (Blanton et al., 2015).

In SHR, long-term administration of *L. fermentum* or *L. coryniformis* plus *L. gasseri* (3.3 × 10^10^ CFU/day, for 5 weeks) similarly induced a progressive reduction in the systolic arterial pressure without significant modifications of the HR (Gómez-Guzmán et al., 2015). This finding was linked to improved endothelial function, reduced vascular oxidative stress and decreased vascular inflammation in the aorta of SH rats (Gómez-Guzmán et al., 2015). Interestingly, recombinant *L. plantarum* expressing angiotensin converting enzyme inhibitory peptide was effective in the diminution of BP in SHR. This finding was linked to increased levels of NO, as well as decreased levels of endothelin and angiotensin II in plasma, heart, and kidney in SHR (Yang et al., 2015).

The treatment with the probiotic formulation termed VSL#3 (*Streptococcus thermophilus, B. longum, B. breve, B. infantis, L. acidophilus, L. plantarum, L. casei, L. bulgaricus*) prevented endothelial dysfunction and improved vascular oxidative stress most likely by reducing bacterial translocation and the local angiotensin system in the mesenteric artery of rats with portal hypertension (Rashid et al., 2014). Another mechanism involved in the antihypertensive effect of probiotics is the production of bioactive peptides with angiotensin converting enzyme (ACE) inhibitory properties during the fermentation process (Thushara et al., 2016). ACE inhibition, in turn, lowers the synthesis of angiotensin II, which result in attenuation of vasoconstriction and blood pressure.

## Clinical findings

Initial clinical testing of the hypothesis that probiotics could reduce BP has been performed in small-scale, double blind, placebo-controlled studies. For example, supplementation of the diet with *L. plantarum* for 6 week in a population of smokers of both genders resulted in reduced systolic BP, improvement of metabolic alterations and attenuated generation of reactive oxygen species (Naruszewicz et al., 2002). Nevertheless, in an investigation in postmenopausal women with metabolic syndrome, a 14 days supplementation with fermented or non-fermented milk supplemented with *L. plantarum*, did not result in an improvement of systolic or diastolic BP. However, a significant reduction in total cholesterol, low-density protein cholesterol, glucose, homocysteine and inflammatory biomarkers was observed (Barreto et al., 2014), although this was not associated to reduced arterial BP, perhaps because of the short duration of the study. Similarly, administration to obese hypertensive patients of an hypocaloric diet (1500 kcal/day), supplemented with cheese containing the probiotic *L. plantarum* showed a remarkable reduction of body mass index associated with decrease of BP when compared to a control group receiving the same diet without probiotic supplementation (Sharafedtinov et al., 2013).

In a randomized double-blind clinical trial on type 2 diabetic volunteers, the supplementation with probiotic soymilk (containing *L. planetarium* A7) did not change the anthropometric parameters (represented by body mass index and waist to hip ratio), however, it reduced both systolic and diastolic BP (Hariri et al., 2015).

On the other hand, a 6-weeks randomized, controlled, parallel, double blind, factorial study performed in overweight men and women, demonstrated that consumption of *L. acidophilus* and *B. animalis* (at a dose of 3 × 10^9^ CFU/day) did not significantly alter BP, HR, total cholesterol, lox density lipoprotein, high density lipoprotein, or triglycerides (Ivey et al., 2015). Similarly, another double-blind randomized controlled study, found that *L. acidophilus* and *B. bifidum* supplementation did not reduce systolic or diastolic BP in healthy adults with hypercholesterolemia (Rerksuppaphol and Rerksuppaphol, 2015). These findings suggest that the choice of an appropriate strain, as also the optimal dosage is crucial to achieve ideal beneficial effects from probiotics.

## Conclusion

The experimental and clinical findings summarized in this review suggest that dietary polyphenols or probiotic consumption may reduce BP and improve cardiovascular risk markers. Future studies investigating the effects of different polyphenolic compounds and probiotics, optimal dosage, intervention times, and studies on the underlying molecular mechanisms leading to improved control of BP are recommended to clarify the beneficial effects of dietary polyphenols and probiotics on AH. In addition, studies are needed to investigate the combined supplementation with dietary polyphenols and probiotics on the BP levels.

## Author contributions

JB and LP drafted the work and revised critically for important intellectual content, wrote the paper, and performed the final review of the manuscript. Vd, MC, MM, VB, JC, CL, and HV contributed to the conception of the work and performed the final review of the manuscript.

### Conflict of interest statement

The authors declare that the research was conducted in the absence of any commercial or financial relationships that could be construed as a potential conflict of interest.
